# Cell Culture Replication of a Genotype 1b Hepatitis C Virus Isolate Cloned from a Patient Who Underwent Liver Transplantation

**DOI:** 10.1371/journal.pone.0023587

**Published:** 2011-08-24

**Authors:** George Koutsoudakis, Sofia Perez-del-Pulgar, Mairene Coto-Llerena, Patricia Gonzalez, Jakub Dragun, Laura Mensa, Gonzalo Crespo, Miguel Navasa, Xavier Forns

**Affiliations:** Liver Unit, Institut D'Investigacions Biomèdics August Pi i Sunyer, Centro de Investigación Biomédica en Red: Enfermedades Hepáticas y Digestivas, Hospital Clínic, Barcelona, Spain; Chungbuk National University, Republic of Korea

## Abstract

The introduction of the genotype 2a isolate JFH1 was a major breakthrough in the field of hepatitis C virus (HCV), allowing researchers to study the complete life cycle of the virus in cell culture. However, fully competent culture systems encompassing the most therapeutically relevant HCV genotypes are still lacking, especially for the highly drug-resistant genotype 1b. For most isolated HCV clones, efficient replication in cultured hepatoma cells requires the introduction of replication-enhancing mutations. However, such mutations may interfere with viral assembly, as occurs in the case of the genotype 1b isolate Con1. In this study, we show that a clinical serum carrying a genotype 1b virus with an exceptionally high viral load was able to infect Huh7.5 cells. Similar to previous reports, inoculation of Huh7.5 cells by natural virus is very inefficient compared to infection by cell culture HCV. A consensus sequence of a new genotype 1b HCV isolate was cloned from the clinical serum (designated Barcelona HCV1), and then subjected to replication studies. This virus replicated poorly in a transient fashion in Huh7.5 cells after electroporation with *in vitro* transcribed RNA. Nonetheless, approximately 3 weeks post electroporation and thereafter, core protein-positive cells were detected by immunofluorescence. Surprisingly, small amounts of core protein were also measurable in the supernatant of electroporated cells, suggesting that HCV particles might be assembled and released. Our findings not only enhance the current method of cloning *in vitro* HCV replication-competent isolates, but also offer valuable insights for the realization of fully competent culture systems for HCV.

## Introduction

Hepatitis C virus (HCV) is an enveloped positive-strand RNA virus that belongs to the *Flaviviridae* family [1]. HCV infection is a major cause of chronic hepatitis, and can lead to liver cirrhosis and hepatocellular carcinoma in a significant percentage of infected patients. Current interferon-α based therapy, in combination with ribavirin, has limited efficacy and is often beset with side-effects [2]. A better understanding of the virus life cycle could be helpful for the development of new therapies, especially for the drug-resistant genotype 1b.

Since hepatitis C virus was first identified in 1989 as the major cause of non-A and non-B hepatitis [3], great progress has been made, both in understanding the molecular basis of the virus and in elucidating the distinct stages of its viral life cycle. Among the milestones achieved in HCV research thus far are the heterologous expression systems [4], [5], the infection of the animal chimpanzee model (*Pan troglodytes*) by HCV cDNA clones [6], [7], the subgenomic replicon system [8], the HCV pseudoparticle system (HCVpp) [9], [10], and finally those cell culture systems based on the JFH1 isolate, which facilitates comprehensive study of the viral life cycle *in vitro* [11]–[14].

The HCV genome consists of the 5′ non-translating region (5′ NTR), a single open-reading frame encoding at least 10 proteins, and the 3′ NTR. The viral particle is composed of the structural proteins, the core (C), and the envelope glycoproteins (E1 and E2). The other non-structural proteins (NS proteins) include the viroporin ion channel p7, the NS2-3 protease, the NS3 dual-function protein (serine protease and helicase), the NS4A polypeptide, the NS5A phosphoprotein, and the NS5B RNA-dependent RNA polymerase (RdRp) [15]. Replication of HCV in cultured human hepatoma cells remains an ongoing challenge to researchers. The first autologous-replicating HCV molecules, described in Lohmann *et. al.* [8], were based on the genotype 1b isolate Con1 and required “replication-enhancing mutations” (REMs) within the NS proteins in order to increase RNA replication to levels sufficient for experimental analyses [16]. Efficient replication of subgenomic replicons without the need for REMs was achieved with the introduction of the JFH1 isolate [17]. Full-length *in vitro* transcribed RNA of JFH1, if introduced to the hepatoma cell line Huh7 via electroporation, leads to the formation of a cell culture system that encompasses both the extracellular and intracellular stages: translation of the RNA, formation of replication complexes for the production of nascent positive- and negative-strand RNAs, generation of new viral particles and secretion, attachment of the new viral particles to neighbouring cells and re-initiation of the cycle. Nevertheless, (with one exception – the chimeric virus J6/JFH1 [11]), JFH1 and all intra- and inter- genotypic chimeric viruses based on the JFH1 isolate require cell culture adaptive mutations that increase virus titers without affecting replication [18]–[22].

Production of infectious genotype 1a HCV in cultured hepatoma cells has also been described in the case of the H77 isolate containing 5 cell culture adaptive mutations (H77-S) [23]. Although this system overcame the genotype obstacle which had plagued HCV cell culture systems, until recently it has found very few applications. Persistent growth of a human plasma-derived hepatitis C virus genotype 1b isolate in cell culture has also been described. This system, however, is based on VeroE6 cells, which contain a homozygous deletion of the IFN-α/β genes. Moreover, HCV replication is enhanced by the adenovirus-associated RNA_I_ (VA-RNA_I_), thus rendering the system inappropriate for immune studies that involve ongoing HCV infections [24].

Here, we exploited the ability of a HCV genotype 1b clinical serum to infect the hepatoma cell line Huh7.5. For this purpose, we used a serum sample with an exceptionally high viral load, obtained from a patient with recurrent hepatitis C following liver transplantation (LT). Viral RNA and HCV proteins were detected 4 days post inoculation and the infection occurred in a CD81-dependent manner. In addition, we constructed a consensus full-length HCV cDNA clone from this serum, designated Barcelona HCV1 (BHCV1), and carried out *in vitro* replication studies in Huh7.5 cells. Finally, in further analyses we generated an inter-genotypic chimeric virus between BHCV1 and JFH1.

## Results

### Inoculation of Huh7.5 cells by a clinical serum

A clinical serum was prepared based on a sample provided by a chronic HCV patient who had undergone LT and who presented severe and recurrent hepatitis with an exceptionally high viral load (for detailed information see the *Materials and Methods* section). We chose the “cured” Huh7 replicon cell clone designated Huh7.5 [25] for our replication and infection studies due to its ability to support high levels of replication and also because it expresses sufficient levels of diverse HCV receptors on the surface [26].

**Figure 1:**
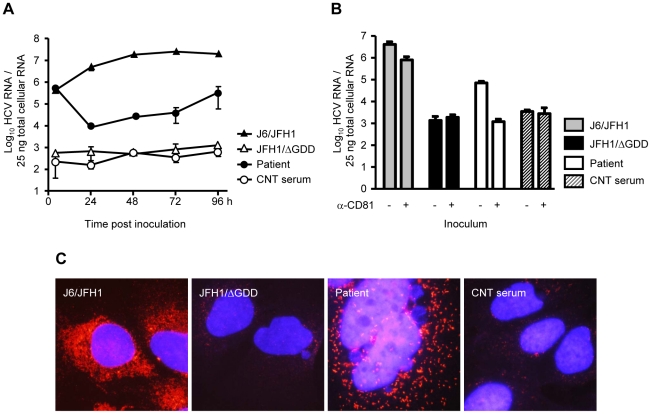
HCV RNA and core protein detection in Huh7.5-inoculated cells. (A and B) HCV RNA was analyzed by qRT-PCR analysis at the indicated time points (A) or at 96 h post inoculation (B). Results represent the mean values from duplicate wells, measured in triplicate, from a representative experiment of 3 (mean ± SD; n = 6). (C) Cells were stained with α-core specific antibodies (clone C7-50) and α-mouse Alexa-568 antibodies (red). Cell nuclei were counterstained with DAPI (blue), magnification 100×.

Initially, Huh7.5 cells were inoculated with the patient serum; additionally, as a negative control, cells were inoculated with a control HCV negative serum. As a reference for our inoculations we used the following cell cultured prepared viruses (HCVcc): J6/JFH1 as a positive control and supernatant from cells electroporated with a replication-deficient form of the JFH1 isolate (JFH1/ΔGDD) as a negative control. HCV RNA extracted from inoculated cells was analyzed by specific quantitative reverse-transcription PCR (qRT-PCR). Analysis was conducted in a time-course fashion in order to better understand the HCV replication. As shown in Fig. 1A, 4 h post inoculation with patient serum, HCV RNA detection was as efficient as that achieved with infection by J6/JFH1 HCVcc inoculation. However, these serum viruses presented much lower replication capacities in Huh7.5 cells than did the J6/JFH1 viruses based on the replication curve calculated at 96 h post inoculation. In addition, we performed neutralization studies with a well-characterized α-CD81 receptor antibody. To this end, cells were incubated with either the neutralizing α-CD81 antibody JS-81 or mock incubated, one hour prior to inoculation. Afterwards, cells were inoculated with diverse viruses. Analysis of HCV RNA was performed 96 h post inoculation. As shown in Fig. 1B, both infections of Huh7.5 cells – that by J6/JFH1 and that by serum viruses – were effective and were neutralized by α-CD81 antibodies. Nevertheless, infection with the patient serum was much less efficient than that recorded with the J6/JFH1 infection, as was similarly observed in the replication-kinetic experiment (Fig. 1A); in the case of the J6/JFH1 virus an MOI 0.2 TCID_50_/mL was used, whereas the MOI for the serum infection was 100 IU/mL. However, the distinct MOI estimation methods used for HCVcc and the serum-derived virus could have caused a discrepancy since the COBAS Taq-Man HCV Test cannot discriminate between functional and non-functional HCV particles and free serum HCV RNA. Our efforts to eliminate background HCV RNA measurements below ∼10^3^ molecules/25 ng total RNA were unsuccessful, probably due to traces of genomic DNA still present in the final sample preparation.

To further corroborate our findings, we performed immunofluorescence (IF) staining of core protein in inoculated cells (Fig. 1C). Core-positive cells for J6/JFH1-infected cells were detectable as early as 24 h post infection, whereas core-positive cells for serum-inoculated cells were present from 72 h post inoculation and thereafter. IF staining 96 h post inoculation was substantially more intense in the J6/JFH1-infected cells. Most likely, the less intense core signals detected in serum-inoculated cells reflect of lower RNA replication, as deduced by the qRT-PCR data, than of the antigenic differences inherent in the α-core antibodies and of the distinct half-life periods found in the core molecules.

### HCV cDNA consensus clone isolated from patient serum

**Figure 2:**
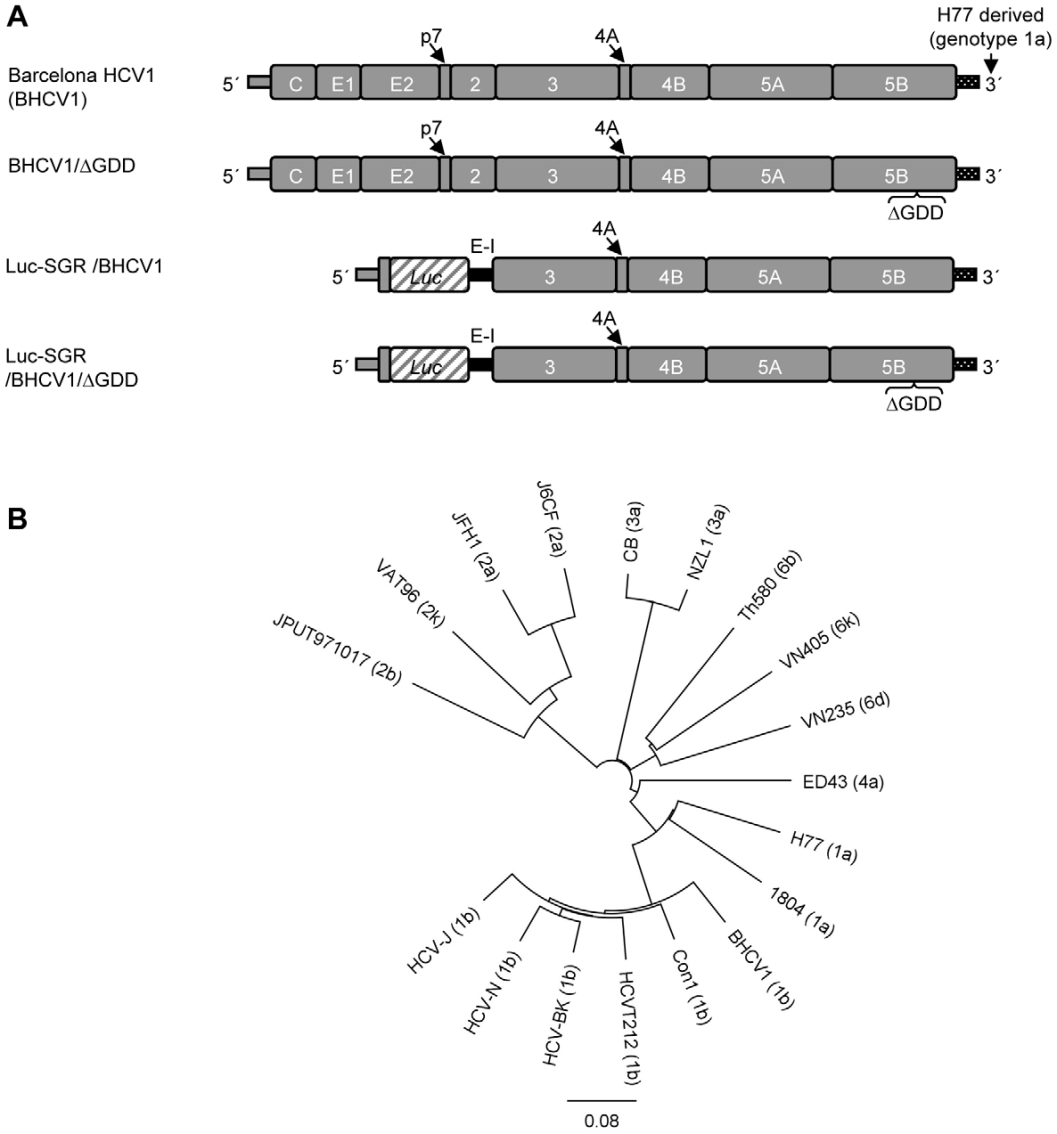
Presentation of the Barcelona HCV isolate 1. (A) Genetic structure of the isolate Barcelona HCV1 (BHCV1), the subgenomic variant (Luc-SGR) with *firefly luciferase* reporter gene (Luc) and the negative control viruses with a GDD deletion (ΔGDD) at the NS5B region (EI: Encephalomyocarditis virus). (B) A phylogenetic tree drawn of the BHCV1 isolate including strains of various genotypes (3′ NTRs were excluded from this analysis). JFH1 accession number is AB047639; HC-J6CH is D00944; VAT96 is AB031663; ED43 is GU814265; H77 is AF009606; 1804 is AM910652; Con1 is AJ238799; HCV-J is D90208; HCVT212 is AB049099; HCV-N is S62220; HCV-BK is M58335; VN235 is D84263; Th580 is D84262; VN405 is D84264, CB is AF046866; JPUT971017 is AB030907 and NZL1 is D17763; The length of the horizontal bar indicates the number of nucleotide substitutions per site.

A full-length consensus HCV cDNA was drawn from the serum by cloning the 5′ NTR with a 5′/3′ RACE commercial kit and the full open-reading frame with 5 overlapping PCRs (see *Supporting Information*). The full-length clone genotype 1b was completed by adding the 3′ NTR of the genotype 1a H77 isolate (Fig. 2A). The addition of a heterologous 3′ NTR fulfilled the prerequisite for a kissing-loop interaction between the stem loop 5BSL3.2 at the NS5B-coding region and the stem loop X-tailSL2 at the X-tail of the 3′ NTR as described in Friebe *et. al.* [27] (data not shown). This new isolate, designated Barcelona HCV1 (BHCV1), possesses a 9591 nt genome. BHCV1 also contains a long open-reading frame spanning nt 342–9377, and codes 3012 amino acids (aa). The well-characterized genotype 1b isolates Con1 [8] and CG1b [28] encode only 3010 aa. Compared to Con1 and CG1b, BHCV1 isolate contains two extra amino acids in the intergenotypic variable region of E2 (IgVR), as does JFH1 isolate. Figure 2B represents a neighbor-joining phylogenetic tree composed of BHCV1 and other known HCV isolates of various genotypes. The tree was created with the Tamura-Nei model [29] and the exclusion of the 3′ NTRs of all isolates was accounted for. The full-length sequence of the BHCV1 isolate has been submitted to the GenBank database under accession number HQ719473.

### Replication studies

In order to perform replication studies with the new cloned BHCV1 isolate, we designed a subgenomic replicon construct carrying the *firefly luciferase* gene as a reporter (Luc-SGR-BHCV1). Translation of the firefly gene occurs under the HCV internal ribosomal entry site (IRES, nt 1–389), and is followed by the heterologous IRES from the encephalomyocarditis virus (E-I) (Fig. 2A). The latter drives the expression of HCV proteins NS3-NS5B, similar to previously described subgenomic replicons [8]. A negative control construct was cloned by deleting the NS5B GDD motif responsible for RNA replication (ΔGDD). These constructs, as well as the full-length BHCV1 construct and its ΔGDD negative control, were cloned into puC vectors downstream of a T7 promoter for the production of *in vitro* RNA transcripts.

**Figure 3:**
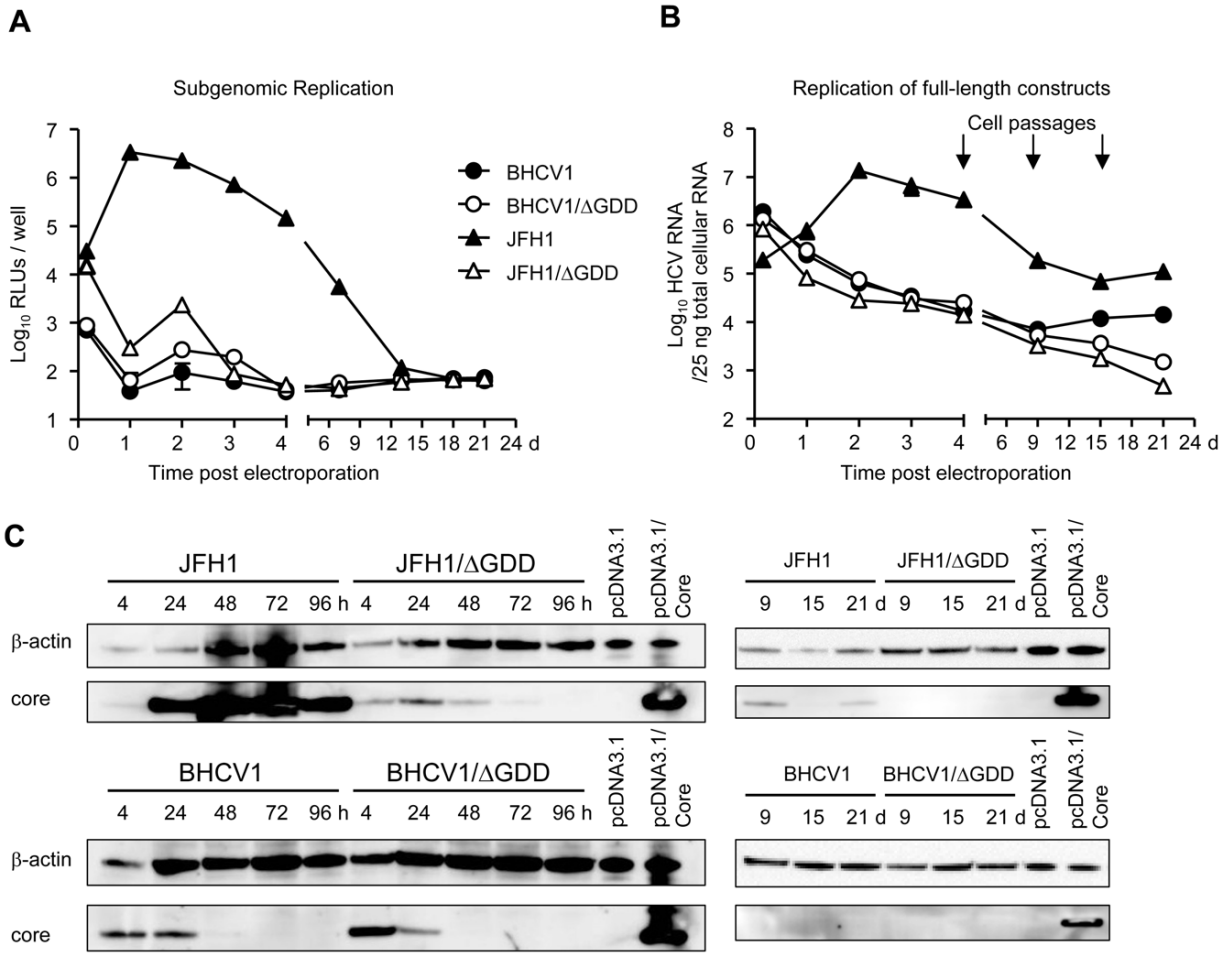
Replication studies of the BHCV1 isolate. (A) Luciferase activity in the electroporated cells with the sugbenomic RNAs in both transient (4 h–4 d) and mid-term fashions (4 d–21 d). (B) HCV RNA in the electroporated cells at similar time points as determined by quantitative reverse-transcription real-time PCR (qRT-PCR). For A and B, data represent the mean values from duplicate wells, each measured in triplicate, from a representative experiment of 3 (mean ± SD; n = 6). (C) Western blot analysis of core protein in electroporated cells with full-length RNAs expressed intracellularly in short-term (4–96 h) and mid-term fashions (9–21 d). Positive and negative control cell lysates were obtained from Huh7.5 cells transfected with a core protein expression plasmid under the control of the CMV promoter (pcDNA3.1/Core) or an empty plasmid (pcDNA3.1), respectively.

Huh7.5 cells were electroporated with RNA from subgenomic replicons of BHCV1, the ΔGDD acting as a negative control, or with equivalent constructs of the JFH1 isolate serving as references. Post electroporation luciferase activity was measured both in a short-term (4 h to 4 d) and in a mid-term fashion (up to 21 d post electroporation), in which cells were passed 3 times (on days 4, 9, 15). As shown in Fig. 3A, significant amounts of luciferase activity can be measured only in cells transfected with the JFH1 construct and then only in a transient fashion. Cells transfected with the Luc-SGR-BHCV1 RNA did not express significant differences in luciferase activity compared to those cells transfected with both negative ΔGDD controls, which suggests that this construct underwent little or no replication.

Huh7.5 cells harboring the subgenomic JFH1 replicon typically undergo a period of cell crisis, beginning just a few days post electroporation, most likely due to the high replication capacity of this isolate [14], [21]. In our studies, Huh7.5 cells electroporated with subgenomic replicons also entered such a crisis phase approximately 9 days post electroporation. JFH1 subgenomic replicons failed to replicate in Huh7.5 either during or after cell crisis (Fig. 3A). In contrast, Huh7.5 cells electroporated with the subgenomic replicons of the BHCV1 isolate did not present any crisis markers or signs of toxicity (data not shown).

In an attempt to obtain a better image of BHCV1 isolate replication, we electroporated Huh7.5 cells with *in vitro* transcripts of full-length virus. Similar to those replication studies involving subgenomic replication constructs, we included a ΔGDD as a negative control and JFH1 constructs as references. RNA replication in the electroporated cells was monitored by quantitative reverse-transcriptase PCR (qRT-PCR), with conserved primers binding at the 5′ NTRs of both isolates (BHCV1 and JFH1). As shown in Fig. 3B, apart from the cells electroporated with JFH1 RNA, in all other cells the HCV RNA gradually decayed during the experiment. The presence of HCV RNA molecules above the cut-off point specified by this method (∼10^3^ molecules/25 ng total cellular RNA) up to 21 days post electroporation can be likened to a slow degradation of the input HCV RNA rather than an active replication of these constructs. Additionally, the JFH1 virus replicated in cells both during and after the cell crisis period, in contrast to the subgenomic replicon, albeit at low levels. Finally, two weeks post electroporation and after 2 cell passages, we detected low amounts of HCV RNA molecules in those cells electroporated with full-length BHCV1 RNA, although still to a significantly higher degree than was the case with the negative controls. This was additionally confirmed for the 21 d post electroporation time-point, which suggests that replication of this virus was attenuated.

**Figure 4:**
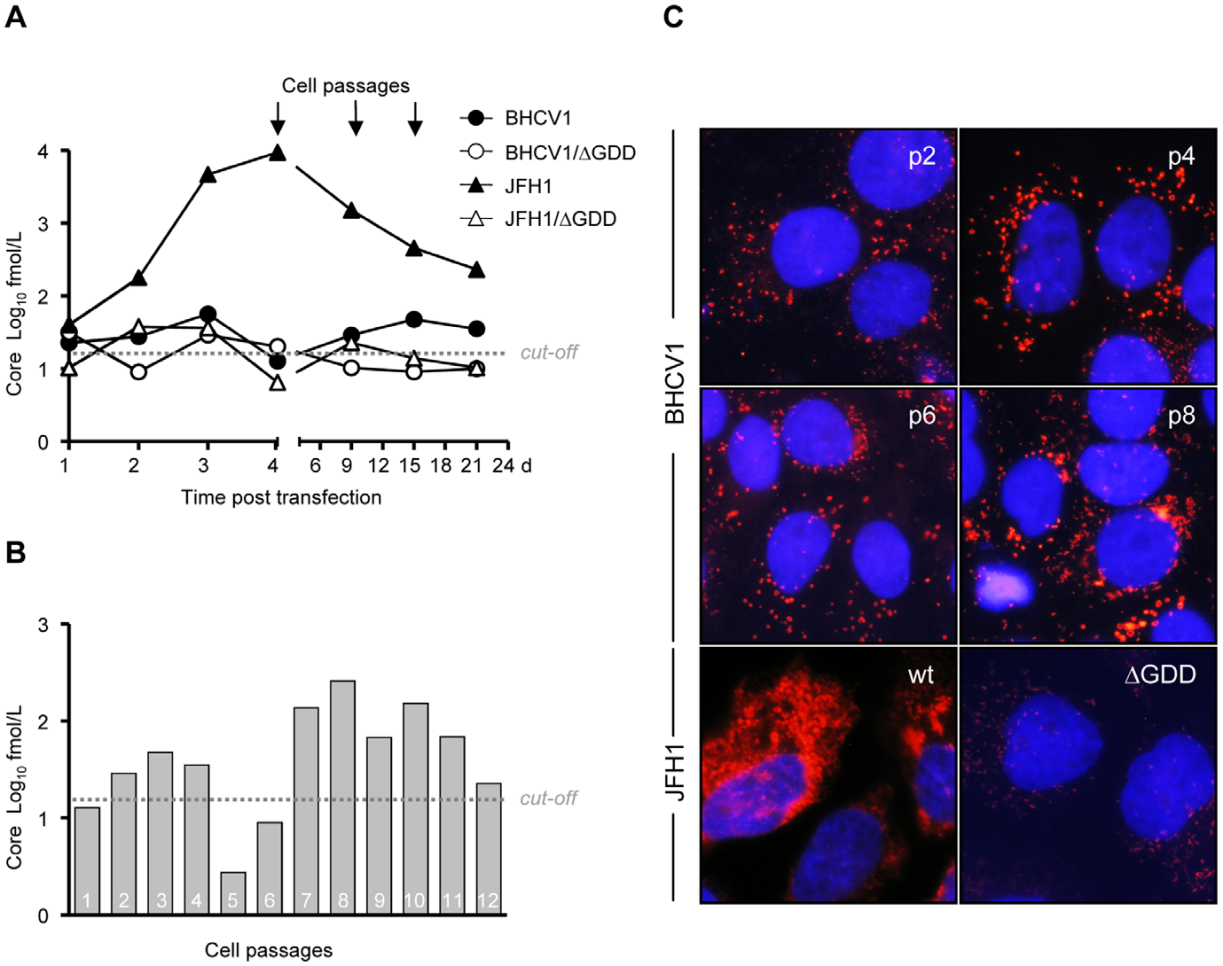
Core protein production during cell passages. (A) Core protein measured in the supernatant of JFH1- or BHCV1-electroporated Huh7.5 cells, and the equivalent ΔGDD negative controls, both in short-term and mid-term fashions. (B) Core protein released in the supernatant from BHCV1 electroporated cells at different passages (C) Intracellular core expression at different passages as detected by core protein IF. Cells were stained with α-core specific antibodies (clone C7-50) and α-mouse Alexa-568 antibodies (red). Cell nuclei were counterstained with DAPI (blue), magnification 100×.

To further corroborate our results, we not only performed Western blot analysis of intracellular core expression in electroporated cells (Fig. 3C), but also quantified core protein expression in the supernatant of the cell cultures (Fig. 4A). As shown in Fig. 3C, transient intracellular core expression was detected in cells transfected with the JFH1 RNA. Core expression decreased at days 9, 15 and 21 post electroporation due to the loss of replication potency. For the BHCV1 construct, an intracellular core was detected as early as 4 to 24 h post electroporation, reflecting the input RNA translation. No core proteins were detected in cell extracts at the remaining time points of the experiment, detections of which continued until 21 d post electroporation.

As shown in Figure 4A, we measured the extracellular core using a core ELISA at these particular time points. Huh7.5 cells transfected with JFH1 RNA presented a significant degree of core release (∼10^4^ fmol/L) at 4 days post electroporation. For all other constructs, including the negative ΔGDD controls, core release was minimal, measuring very close to the cut-off level we set (20 fmol/L) based on our experience with the commercial core ELISA kit. These small amounts of core protein most likely stemmed from the input RNA expression, as was clearly demonstrated by the Western blot analysis. Interestingly, at 24 h post electroporation core levels in the supernatant of the BHCV1-electroporated cells were not elevated, as was the case with the low-replicating Con1 isolate [8], which suggests that some impairment in particle production occurs within the first hours post electroporation. On the other hand, low amounts of core protein could be detected in the supernatant of BHCV1-electroporated cells beginning at 15 d post electroporation. This is in contrast to the core protein levels detected in those cells electroporated with the ΔGDD constructs, which fell below the cut-off level. The latter observation is consistent with the low amount of intracellular RNA detected in BHCV1-electroporated cells 15 d post electroporation and thereafter.

Taken together, we believe that the replication capacity of the BHCV1 isolate remains very low during the first 3 weeks following electroporation of Huh7.5 cells. In fact, it cannot be detected either by intracellular HCV protein analysis or by use of subgenomic replicons carrying luciferase reporters. Only very specific and accurate methods, such as qRT-PCR or core ELISA analysis, enabled us to detect low amounts of RNA molecules or core antigen, respectively.

### Cell passages

Based on our previous results, we tried to adapt the BHCV1 virus in cell culture. To this end, Huh7.5 cells electroporated with BHCV1 virus were passaged up to 12 times. Extracellular core secretion was monitored by core ELISA (Fig. 4B). BHCV1 replication was monitored indirectly by IF analysis of core expression intracellularly, at different cells passages (Fig. 4C). As shown in Fig. 4B, the low core secretion levels presented in passages 1–4 fell below the cut-off limit of the core ELISA for passages 5–6. Nevertheless, starting at passage 7, core secretions to the supernatant elapsed, reaching maximum levels (up to 250 fmol/L) by passage 8. After this time point, core secretions began to decline, although they remained quite detectable. In similar fashion, intracellular core expression peaked around passage 8, with core-positive cells constituting at least 20% of the total cell population (Fig. 4C). Nonetheless, intracellular core expression levels, as well as extracellular core secretion, remained low compared to that produced by the JFH1 isolate.

**Figure 5:**
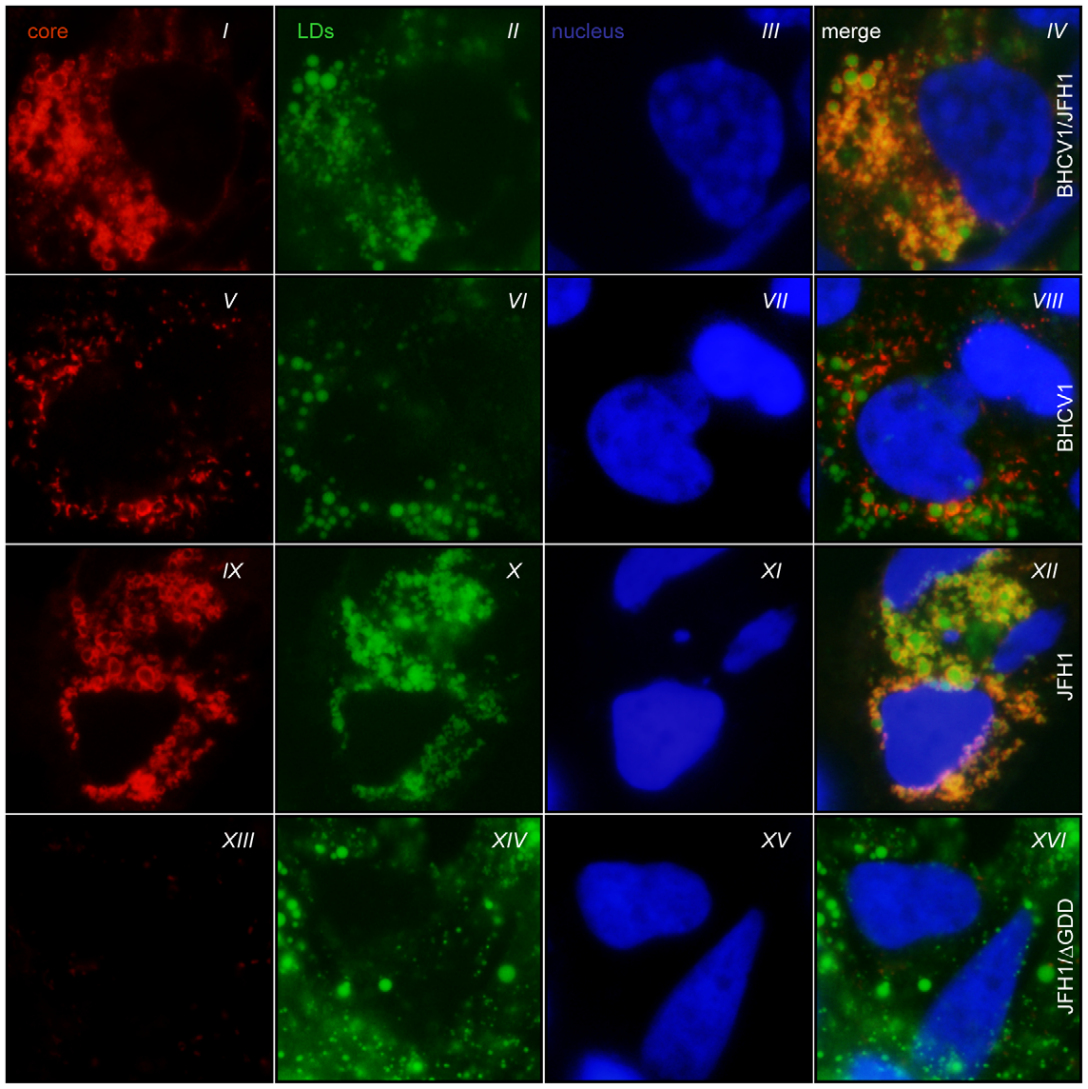
Core protein subcellular localization on LDs. Huh7.5 cells were electroporated with BHCV1/JFH1 (panels *I* to *IV*), JFH1 (panels *IX* to *XII*), JFH1/ΔGDD (panels *XIII* to *XVI*) and fixed 72 h post electroporation. Panels V to VIII represent fixed cells of BHCV1 transfected cells at passage 9. Fixed cells were analyzed for the subcellular localization patterns of core protein (red) by immunofluorescence, whereas LDs were stained with BODIPY493/503 (green). Cell nuclei were counterstained with DAPI (blue), magnification 100×.

To firmly demonstrate the presence of core protein expression within passaged cells, we conducted a detailed immunofluorescence analysis of 1) the core protein expressed in BHCV1-transfected cells at the higher passages and of 2) that expressed in Huh7.5 cells following RNA electroporation of the JFH1 isolate. Core protein within infected and JFH1-electroporated cells partially surrounds the lipid droplets (LDs) wherein assembly of nascent viral particles is believed to take place [30]. We were able to show a clear co-localization of core protein expression with LDs in BHCV1-passaged cells, similar to that observed in JFH1-electroporated cells, although core levels were lower (Fig. 5, panels *V–VIII*). To further corroborate our findings with a virus that expresses high amounts of BHCV1 core protein, we performed the same IF analysis in Huh7.5 cells electroporated with the RNA of a BHCV1/JFH1 intergenotypic chimeric virus (described in detail in the next subsection). Most of the core protein expression within these electroporated cells was also found to surround the LDs (Fig. 5, panels *I–IV*).

Since it is known that HCV in cell culture accumulates cell culture adaptive mutations, resulting in either replication-enhancing mutations [16] or in adaptive mutations that have no effect on replication but that increase virus production or infectivity [18], [19], [21], [22], we sought to extract BHCV1 RNA from passaged cells and sequence the entire genome. To this end, total RNA was prepared from BHCV1 Huh7.5-electroporated cells (passage 10) and by employing the same strategy used for cloning the BHCV1 isolate from patient serum, we amplified the passaged BHCV1 genome. This confirmed the existence of the BHCV1 genome in the passaged cells since re-amplification of the genome from total RNA cell extract was possible. Surprisingly, we mapped only one adaptive mutation in the E2 region (V632I at the amino acid level, GTC→ATC at the nucleotide level), suggesting that the BHCV1 isolate does not accumulate adaptive mutations in cell culture.

### BHCV1/JFH1 intergenotypic chimeric virus

**Figure 6:**
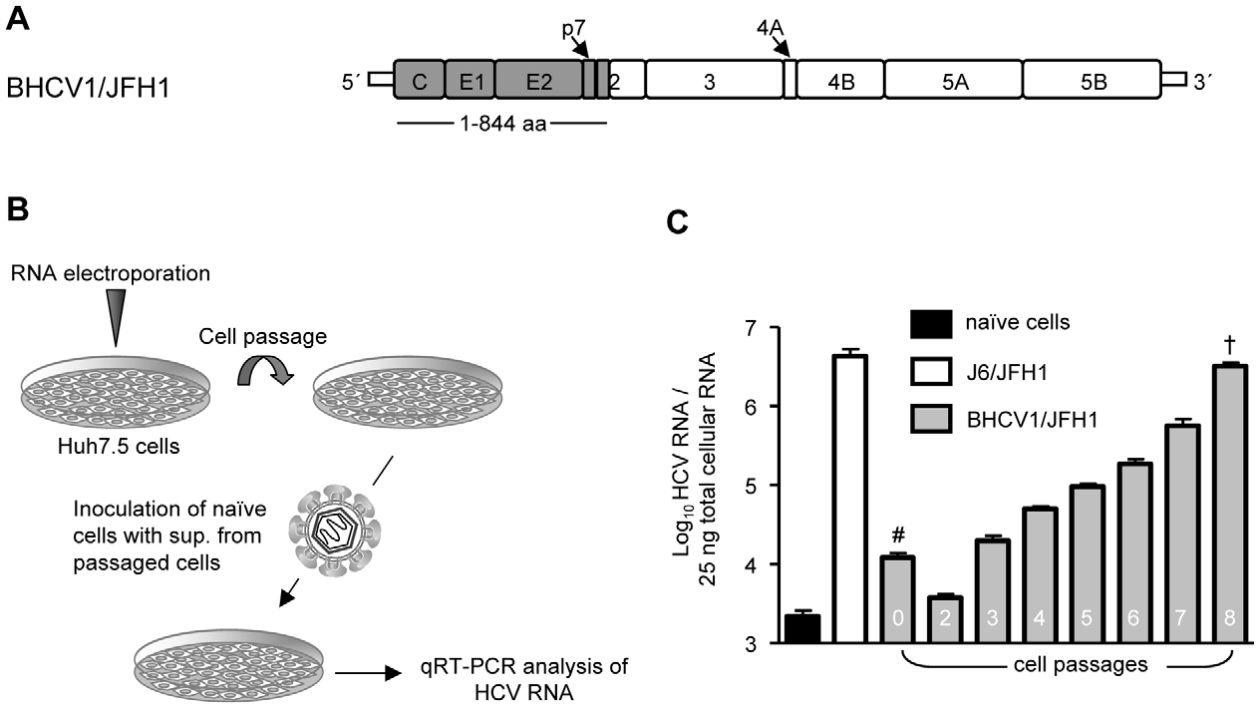
Infectivity of the BHCV1/JFH1 chimeric virus. (A) Genetic structure of the BHCV1/JFH1 chimeric virus. Structural proteins, p7, and the first transmembrane domain of NS2 of the isolate BHCV1 (amino acids 1–844) were fused in-frame to the remaining non-structural proteins of the JFH1 isolate. Both NTRs are of JFH1 origin. (B) Schematic presentation of the experimental plan for the cell culture adaptation of the BHCV1/JFH1 chimeric virus. (C) qRT-PCR analysis of naïve Huh7.5 cells inoculated with supernatant derived from Huh7.5 cells containing the BCHV1/JFH1 virus from different passages. J6/JFH1 infection: MOI 0.2 TCID_50_/mL. Data are expressed as the mean of duplicate infections measured in triplicate (mean ± SD; n = 6). # Estimated infectivity ∼10^2^ TCID_50_/mL; † Estimated infectivity ∼10^5^ TCID_50_/mL.

To further characterize the BHCV1 isolate, we created a BHCV1/JFH1 intergenotypic chimeric virus and subjected it to replication and infection studies (Fig. 6A). Just as inter-genotypic JFH1 chimeric viruses have been conclusively shown to produce low levels of infectious particles [20], so too the BHCV1/JFH1 chimera did produce infectious particles, albeit at low levels (∼10^2^ TCID_50_/mL), specifically at 4 days post Huh7.5 cell electroporation with *in vitro* RNA transcripts. These cells (passage 0) were passaged, with cell culture supernatant being tested each time for its infectivity against naïve Huh7.5 cells (Fig. 6B). As shown in Fig. 6C, at each passage, up to passage 8, cell culture supernatants contained higher amounts of infectious virus, reaching infectivity titers similar to those observed with the high-virus-productive J6/JFH1 chimera (∼10^5^ TCID_50_/mL), suggesting that the chimera BHCV1/JFH1 is prone to cell culture adaptation. We additionally verified this finding with α-core IF analysis comparing an enhanced virus spread of the adapted virus with the initial chimera (data not shown).

Total RNA from Huh7.5 cells (passage 8) was extracted, cDNA was prepared, and then both parts of the virus, the BHCV1 and the JFH1, were cloned and sequenced. In the case of BHCV1, we mapped one adaptive mutation in the E1 region (C307R) and ten adaptive mutations at the E2 gene: N411D, S467A, A476N, P478S, S479H, D480S, L481S, Q493R, S523F, and T529S (Table 1). Five of these adaptive mutations (positions 476, 478, 479, 480, and 481) were located within the hypervariable region 2 (HVR2) [31], which has been postulated to play a role in the binding of various cell-surface receptors [32]. For the JFH1 part, we identified 1 adaptive mutation at the viral protease NS2 (G899E), 2 at the NS4B protein (E1874Q, V1896G) and 5 at the NS5A phosphoprotein (E2369K, E2383K, D2399N, E2408K, and L2438V). The NS2 amino acid G899 is located at position 88, which is at the end of the protease's third putative transmembrane domain. Changing glycine to leucine at this position had a rather moderate inhibitory effect on particle production in the J6/JFH1 chimeric virus [33]. The two mutations identified at the NS4B protein are of special interest since both appeared at very well-conserved amino acid positions. Furthermore, the first 4 mutations found in the NS5A region were clustered in domain III of the molecule, which plays an essential role in HCV particle assembly [34]. Finally, in order to enhance viral assembly and release, we initially introduced the V2440L adaptive mutation at the C terminal domain of NS5A, which improved viral production in the Con1/JFH1 chimeric virus [21]. To our surprise, this mutation reverted to wild-type (L2438V).

**Table 1:** Amino acids substitutions identified in adapted viruses.

Virus	Substitution frequency^a^	Amino acid substitution^b^	Protein
BHCV1	4/4	V632I	E2
BHCV1/JFH1	4/4	C307R	E1
	4/4	N411D	E2
	4/4	S467A	E2
	4/4	A476N	E2
	4/4	P478S	E2
	4/4	S479H	E2
	4/4	D480S	E2
	4/4	L481S	E2
	4/4	Q493R	E2
	4/4	S523F	E2
	4/4	T529S	E2
	2/4	G899E	NS2
	4/4	E1874Q	NS4B
	2/4	V1896G	NS4B
	1/4	E2369K	NS5A
	4/4	E2383K	NS5A
	4/4	D2399N	NS5A
	4/4	E2408K	NS5A
	4/4	L2438V	NS5A

a Frequency of amino acids substitution identified in cloned PCR products.

b Numbers refer to amino acid positions of each respective virus.

### Enhancement of viral entry by gps-adaptive mutations

**Figure 7:**
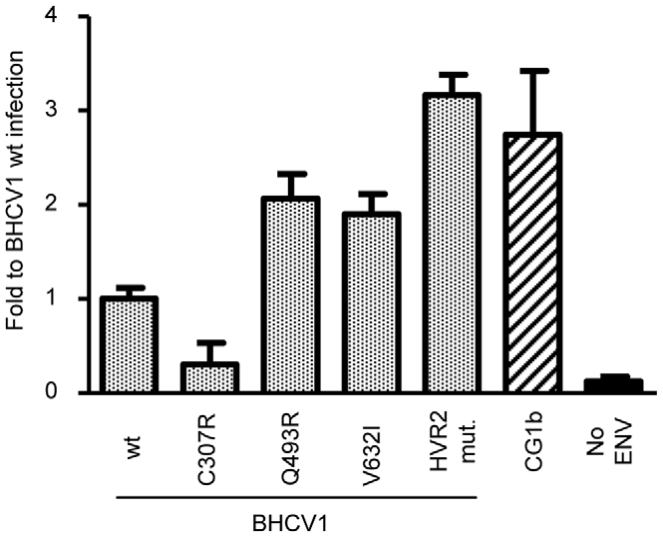
Adaptive mutations enhance infectivity of HCVpp. Infectivity of HCVpp from the BHCV1 isolate harboring adaptive mutations on their surface is indicated; numbers of the amino acids refer to positions at the BHCV1 isolate; “HVR2 mut.” contains 5 mutations clustered at the hypervariable region 2 of the E2 glycoprotein. HCVpp harboring on their surface E1E2 glycoproteins of the CG1b isolate were used as an infectivity control. Infections by pseudoparticles produced without envelope glycoproteins “No ENV” were used as an unspecific infectivity control. Results show a representative experiment of 3. The infectivity is expressed as the mean of fold induction relative to the infectivity obtained from HCV pseudoparticles harboring the wild-type BHCV1 E1E2 glycoproteins. Infections were carried out in duplicate and measured in triplicate (mean ± SD; n = 6).

In order to clarify what role(s) the adaptive mutations in glycoprotein regions may play in the adaption process of the BHCV1 isolate and its chimera with the JFH1 isolate, we generated wild-type and mutated HCV pseudoparticles (HCVpp) for the BHCV1 isolate as follows, with relevant results noted: i. HCVpp bearing the wt E1E2 proteins and, as an infectious control, wt E1E2 from the genotype 1b isolate CG1b [28]; ii. HCVpp harboring E1E2 glycoproteins with the adaptive mutation V632I on their surface appeared in the passaged BHCV1; iii. HCVpp harboring the Q493R mutation; iv. HCVpp harboring all five adapted mutations appeared at the HVR2 region (HVR2 mut) of the chimeric BHCV1/JFH1 virus; and finally, v. HCVpp with the adaptive mutation appeared in the E1 glycoprotein (C307R). Infection studies with the pseudoparticles revealed a 2- to 3-fold enhancement in HCVpp infectivity in those harboring the adaptive mutations versus their wt counterparts (Fig. 7), with the exception of the adaptive mutation C307R. Further reverse-genetic studies should be carried out in order to determine the role(s) played by the other adaptive mutations in entry process of the BHCV1 isolate.

## Discussion

In this study we exploited the potential capacity of a natural HCV virus, present in the serum of a chronically infected patient who had undergone LT, to infect Huh7.5 cells *in vitro* and successfully replicate within these cells. Studies examining HCV kinetics, both during and immediately following LT, have shown that although a rapid drop in HCV viral load occurs during the initial hours after liver graft reperfusion, the viral load then increases as early as 12 h after graft reperfusion. In a significant portion of HCV patients who undergo LT, pre-transplantation levels are surpassed within 1 to 3 months [35]. Here, we used a serum harvested 3 months post LT from a patient with aggressive and recurrent hepatitis C following surgery, and whose condition was characterized as acute cholestatic hepatitis. Both innate and adaptive immunity play important roles in the control of HCV infection. In the transplant setting, however, any anti-HCV response takes place (in most cases) in the context of a non-self histocompatibility complex, in conjunction with severe immunosupression [36]. Revie D. *et. al.*, have reported the transmission of patient-derived HCV in secondary cells (e.g., macrophages, T-cells and B-cells) during long-term cell culture [37]. According to their findings, only patient sera that contain high levels of HCV RNA have sufficient infectious virions to ensure successful transmission of HCV in *vitro*. Therefore, we concluded that our particular serum, with its extraordinary high viral load, is of high value.

Since the discovery of HCV in 1989, researchers have tried to inoculate cells *in vitro* using infectious HCV isolated from HCV-positive patients. These efforts included not only several types of liver cells, but also cells of non-liver origin, such as HeLa, CEM, H9, Jurkat, Molt 3, Molt 4, U937, P3HR1, Raji, and Daudi cells [38]. Unfortunately, the results of these studies, in particular the sustained replication of HCV, remained irreproducible until the JFH1 isolate-based HCVcc system and the highly permissive replication clone Huh7.5 were described [11], [13], [14]. Here, we report that inoculation of Huh7.5 with natural virus causes productive infection, which can then be neutralized by α-CD81 antibodies. Nevertheless, such an infection proved much less efficient than that involving cell culture-produced recombinant HCV virus. Moreover, the natural virus contains a wide variety of quasi-species circulating in the blood of infected patients, thus creating a heterogeneity that greatly hindered further experimental analyses; e.g., entry studies examining various E2 hypervariable regions, etc.

A consensus genotype 1b HCV cDNA clone was reconstructed from patient serum and designated Barcelona HCV1 (BHCV1). Our replication studies with the *firefly luciferase* gene revealed that only the JFH1 genome boasted a high replication capacity, albeit in a transient fashion. Due to the poor and transient replication of the Luc-SGR-BHCV1 construct, we undertook similar efforts with those sub-genomic replicons that carry a *neomycin*-resistant selective marker (Neo-SGR-BHCV1), similar to that described by Lohnmann *et. al* [8]. Unfortunately, we were unable to obtain any *neomycin*-resistant colonies after 21 days of selection with the antibiotic (data not shown), suggesting that this Neo-SGR-BHCV1 construct failed to accumulate any cell culture adaptive mutations. We therefore concluded that although subgenomic replicons have proven to be valuable tools for replications analyses of numerous flaviviruses and could contain various well-tolerated heterologous elements (gene reporters, heterologous IRES etc), comprehensive imaging of a flavivirus' replication capacity can only be obtained with studies that utilize wild-type isolates. Indeed, when Huh7.5 cells entered the “cell crisis” phase, we failed to detect any luciferase activity in the subgenomic replicon, whereas we were able to measure JFH1 replication for the wild-type virus using qRT-PCR. These data suggest that only extremely precise techniques are suitable for measuring the replication capacity of viruses that replicate at low levels, especially for such isolates as BHCV1.

Since replication studies with subgenomic replicons revealed that the BHCV1 isolate had a low *in vitro* replication capacity, we exploited its potential capacity for adaptation by passaging electroporated Huh7.5 cells. As shown in Fig. 4B, core secretions in the supernatant of electroporated cells remained low for the first 6 passages. However, beginning with passage 7 and for 5 passages thereafter, Huh7.5 cells produced sizeable amounts of core secretions, suggesting the presence of particles in the supernatant of the passaged cells. Intracellular core analysis by IF confirmed the presence of core secretions within the passaged cells, which indicated that the BHCV1 isolate now had an increased replication capacity, thus facilitating the ready detection of core protein, both intracellularly and extracellularly. Finally, higher magnification of core-stained cells at passage 9 showed the characteristic circular fluorescence staining around the lipid droplets [30], thus strengthening our hypothesis that the BHCV1 isolate was successfully replicating within these cells. Total RNA was extracted from these cells and then the BHCV1 isolate was amplified, confirming its presence therein. In attempting to sequence this passaged version and to map these cell culture-adapted mutations, we failed to identify any mutations except that located at the E2 region.

These are very important findings for two reasons: First, these results support the hypothesis that a consensus HCV genome can be maintained in passaged cells, although replication capacity does become attenuated. Second, this genome accumulated neither replication-enhancing mutations, which could have been deleterious for particle production as was the case with Con1, nor cell culture-adaptive mutations that might enhance virus production. Therefore, we would suggest that there exist qualitative differences in the manner in which this consensus genome replicates in cell culture that might better reflect the *in vivo* setting.

When we extended our analysis, by creating a BHCV1/JFH1 chimeric intergenotypic virus, we uncovered the expected adapted mutations: the elevation of virus titers to levels, similar to that observed with the chimera J6/JFH1, was also accompanied by adaptive mutations clustered together in the glycoprotein regions. Using reverse genetics, we analyzed the impact of these adaptive mutations on the entry of the BHCV1 isolate, which we accomplished by generating wild-type HCVpp of this particular isolate as well as that HCVpp harboring the adaptive mutations. One of these mutations was mapped to the E1 glycoprotein on a cysteine residue, which is critical for the secondary structure for this glycoprotein. The infection capacity of those HCVpp harboring only this mutation was significantly downgraded due to a loss in stabilization of the covalent complexes formed in association with E2, which are stabilized by disulphide bonds [39]. Further analyses of this mutation, in combination with the other adaptive mutations, should be carried out in order to clarify the role of this particular amino acid change.

As five of the adaptive mutations that arose in the BHCV1/JFH1 chimeric virus appeared in a row clustered along the HVR2 region of the E2 glycoprotein (P478S, S479H, D480S, and L481S), we therefore analyzed all of these mutations in one single mutant HCVpp. As shown in Fig. 7, HCVpp harboring these mutations presented a 3-fold greater infectivity level compared with their wt counterparts. Additionally, we examined the single-point mutation Q493R after observing a turn from a non-polar (glutamine) to a polar amino acid (arginine) arrangement. HCVpp harboring this mutation were 2-fold more infectious than the wt pseudoparticles. Finally, we examined the single mutation that had appeared in the BHCV1 full-length isolate (V632I) and similarly found a 2-fold increase in infectivity in the HCVpp system.

Mutations that arose in the JFH1 part of the BHCV1/JFH1 are of special interest: First, because several conserved amino acids had undergone mutations (i.e., V1896G). Second, due to these amino acids changes, the physicochemical properties of the relevant protein domains most likely changed as well. For example, the G899of NS2, which is a non-polar amino acid and which, according to the NS2 3D structure model, forms part of the third transmembrane domain of NS2 [33], changed to the polar amino acid E. Additionally, 3 polar E of the domain III of NS5A, which has a negative side-chain charge, changed to polar K and had a high positive side-chain charge, confirming the essential role it plays in this region in terms of virion production and assembly. To attempt a reverse-genetics transformation of these NS adaptive mutations back to the wild-type virus remains well beyond the scope of this study. Besides, adaptation of the JFH1 virus in cell culture demands considerable flexibility, since each individual adaptation can lead to different adaptive mutations [21], though all with the same result: an increase in particle production and release.

Although *in vivo* infectivity has thus far been conclusively shown for several consensus HCV isolates, including those of genotype 1b, the production of infectious particles in transfected Huh7 cells, or in their derivatives, has remained problematic because of the low replication rates of these isolates. However, replication-enhancing mutations are not the solution, at least not for the genotype 1b isolate Con1, since such mutations cause impairments in particle production [15]. As shown in this study, the BHCV1 isolate not only maintained the low replication capacity of other consensus isolates, but following several cell passages it increased core (and most likely others) protein expression, both intracellularly and extracellularly. Although there is still no formal proof demonstrating that this core protein corresponds to the virus particles, our data suggest that HCV particle assembly has occurred. The various methods of replication enhancement, such as the treatment of transfected cells with kinase inhibitors or with vitamin B6, may improve this *in vitro* HCV cell culture system and thereby provide a favorable tool for both *in vitro* study of the virus and screening of anti-viral drugs.

In summary, we demonstrate that inoculation of Huh7.5 cells with the serum of an HCV-infected patient can facilitate core and RNA detection of HCV in inoculated cells. Although patient sera are an abundant source of HCV, it is of limited use *in vitro*, most likely due to the quasi-species nature of the virus and because of the low replication capacities of natural viruses in Huh7.5 cells. Thus, the cloning of recombinant HCV isolates could offer one solution for the *in vitro* propagation of the virus. As with many other recombinant consensus isolates the BHCV1 virus replicated poorly in cell culture. Nevertheless, following several cell passages post electroporation and without the aid of replication-enhancing mutations, both BHCV1 core protein-positive cells and core secretion in the supernatant could be detected, which suggests that the machinery of replication and protein production had been enhanced. We believe that HCV isolates with low replication profiles are tolerated at low states of replication within Huh7.5 cells. The realization that such HCV isolates could enhance their replication capability after a certain point, without the need of replication-enhancing mutations, may not only provide new insights into the workings of current and future HCV isolates, but may also finally give rise to new *in vitro* replication-competent isolates.

## Materials and Methods

### Ethics statement

The Investigation and Ethics Committee of Hospital Clinic Barcelona approved our protocol, which conformed to the ethical guidelines of the 1975 Declaration of Helsinki. Written informed consent was obtained from the patient included in this study.

### Patient profile

HCV positive serum was obtained from a 65-year-old chronic female patient who had undergone LT because of HCV-related cirrhosis. The HCV genotype was 1b, which was determined by restriction fragment-length polymorphism analysis of the 5′ NTR of the HCV genome, as previously described [40]. Following LT, the patient developed acute cholestatic hepatitis; three months after LT the patient reached a bilirubin peak of 19 mg/dl and an ALT of 500 IU/mL. At this point her viral load reached an extraordinary high viral level: >10^9^ IU/mL, as deduced by real-time PCR (COBAS Taq-Man HCV Test, Roche Diagnostics, Mannheim, Germany) and it was therefore decided to use this serum for our experimental analyses. Antiviral treatment was started at the usual dose (pegylated interferon alpha 2b 1.5 µg/kg/week and ribavirin 1000 mg/d). A rapid decrease in ALT and bilirubin levels was observed and the viral load reached SVR. In December 2009 the SVR was confirmed; specifically, the viral load fell below the detection limit (<25 IU/mL), remaining at this level even during manuscript preparation.

### Cell culture and cell lines

Huh7.5 and 293T cells were grown in Dulbecco's modified Eagle medium (DMEM; Invitrogen, Carlsbad, CA) supplemented with 2 mM L-glutamine, non-essential amino acids, 100 U of penicillin per ml, 100 µg of streptomycin per ml, and 10% fetal calf serum (FCS), designated DMEM complete, in an incubator with 5% CO_2_ at 37°C.

### Sera collection, HCV inoculations with serum and HCVcc, and α-CD81 neutralization

The patient's blood was collected in Vacutainer® Rapid Serum Tube (Becton Dickinson, Franklin Lakes, NJ) according to standard hospital techniques and serum was separated after centrifugation at 4000 rpm for 10 min. Serum was aliquoted and kept at −80°C. For inoculation of Huh7.5 with patient sera, Huh7.5 cells were seeded onto 12-well plates 18 h prior to inoculation, 8×10^4^ cells/well. At the day of inoculation sera were thawed gently at 4°C. DMEM complete was aspirated from cells, which were washed 3× with PBS. Sera were diluted 1∶10 to DMEM complete without FCS and the DMEM-sera mix was used to inoculate Huh7.5 cells. Four h post inoculation DMEM-sera mix was aspirated, cells were washed 3× with PBS, and then fed with DMEM complete with FCS for 96 h. In the case of HCVcc inoculations, cells were inoculated with cell culture supernatant derived by RNA (J6/JFH1 or JFH1/ΔGDD)-electroporated Huh7.5 cells. For neutralization with α-CD81 antibodies, cells were incubated with α-CD81 antibodies at a concentration of 2 µg/mL (Clone JS-81, BD Pharmingen™, San Diego, CA) for 1 h prior to inoculation with patient sera or HCVcc.

### HCV RNA extraction from serum and cloning procedures

Protocols for HCV RNA extraction from serum and cloning procedures are provided as Materials and Methods S1. Primers' combinations for the cloning of the BHCV1 and JFH1 viruses are presented in Table S1 and Table S2, respectively. Sequences of all primers used in this study are listed in Table S3 (forward primers) and in Table S4 (reverse primers).

### In vitro transcription

Plasmids carrying BHCV1 and JFH1 constructs were linearized with the *XbaI* enzyme and plasmid DNA was purified with the QIAquick PCR purification kit (Qiagen, Düsseldorf, Germany). Purified DNA was subjected to an *in vitro* transcription reaction with the MEGAscript® T7 kit (Applied Biosystems, Foster city, CA) according to the manufacturer's protocol. RNA from the *in vitro* transcription reaction was purified with the Nucleospin® RNA II kit (Macherey-Nagel, Düren, Germany). RNA integrity was verified by denaturing agarose gel electrophoresis and the concentration was determined by measurement of the optical density at 260 nm. Finally, the RNA was stored at −80°C.

### Electroporation of HCV RNAs

Single-cell suspensions of Huh7.5 cells were prepared by trypsinization of monolayers and subsequent resuspension was conducted with DMEM complete. Cells were washed with phosphate-buffered saline (PBS), counted, and resuspended at 1.5×10^7^ cells per ml in Cytomix [41] containing fresh 2 mM ATP and 5 mM glutathione. Ten µg of *in vitro* transcribed RNA was mixed with 400 µl of the cell suspension by pipetting. Cells were then electroporated and immediately transferred to 10 ml of complete DMEM. Subsequently, the cells were seeded at a density of 3.125×10^5^ cells/cm^2^, which corresponds to 2 ml of the cell suspension per well in a six-well plate. Electroporation conditions were 960 µF and 270 V by using a Gene Pulser Xcell™ system (Bio-Rad, Munich, Germany) and a cuvette with a gap width of 0.4 cm (Bio-Rad).

For the generation of HCVcc stocks (J6/JFH1 virus or JFH1/ΔGDD negative control), 10 mL of electroporated cells were seeded onto 10-cm dishes. Supernatant from the electroporated cells was harvested 96 h post electroporation, cleared by passing them through 0.45-µm-pore-size filters. Supernatants were kept at 4°C for up to 3 weeks without significant loss of infectivity, as determined by TCID_50_ estimation of the virus titer.

### Indirect immunofluorescence

Cells were seeded on glass coverslips in 24-well plates at a density of 8×10^4^ per well 24 h before infection, followed by inoculation with 250 µl of filtered cell culture supernatant containing JFH1 or J6/JFH1 virus or patient serum diluted 1∶10 to DMEM complete without FCS. Four h post inoculation inocula were removed, cells were washed 3× with PBS, and fresh DMEM complete was added to all of the cells. After 96 h, cells were fixed with 4% paraformaldehyde in PBS and permeabilized with 0.5% Triton X-100 in PBS. For electroporated Huh7.5 cells, 250 µl of post-electroporation cell suspension were seeded on similar coverslips and the cells were then cultivated for 72 h. Immunostaining of core protein was performed by using the mouse monoclonal α-core antibody C7-50 (Santa Cruz Biotechnologies, Santa Cruz, CA) at a final concentration of 1 µg/mL in PBS supplemented with 5% bovine serum albumin (BSA). Bound primary antibodies were detected using goat α-mouse antibodies conjugated to AlexaFluor® 568 (Invitrogen, Eugene, OR) at a dilution of 1∶1000 in PBS with 5% BSA. DNA was stained with DAPI (4′, 6-Diamidino-2-phenylindole dihydrochlorid) (Sigma-Aldrich, St. Louis, MO). Finally, cells were washed 3× with PBS and once with water and mounted on glass slides with Fluoromount G (Southern Biotechnology Associates, Birmingham, AL). For LD staining, post-incubation cells containing the secondary AlexaFluor® antibody were incubated with 20 µg/mL BODIPY493/503 (Invitrogen, Eugene, OR) for 10 min, washed 3 timed with PBS, and then counterstained with DAPI.

### Preparation of cell lysates, PAGE, and Western blot analysis

In order to prepare cell lysates, electroporated cells were harvested by removing the growth medium and were then washed with PBS. Cells were trypsinized with 0.25% Trypsin-EDTA solution (Sigma-Aldrich, St. Louis, MO), collected, and centrifuged at 1200 rpm for 5 min. The cell pellet was lysed with 1% Triton X-100 in PBS and clarified by centrifugation at 13000 rpm for 10 min. Finally, debris-free supernatant was kept at −80°C. Total protein in the lysates was quantified with a Lowry protein assay and an equal amount of protein for each sample was diluted into sample buffer (160 mM Tris, pH 6.7, 2% SDS, 700 mM β-mercaptoethanol, 10% glycerol, 0.004% bromophenol blue). Samples were heated at 70°C for 10 min to fully denature proteins and then loaded onto a 4–12% SDS-polyacrylamide gel (NuPAGE®, Novex 4–12%, Bis-Tris Midi gels, Invitrogen, Eugene, OR). Following electrophoresis, proteins were transferred to polyvinylidine fluoride membrane (PerkinElmer, Life Sciences, CA). Blots were blocked overnight at 4°C in blocking solution (5% milk powder and 0.05% Tween 20 in PBS). Incubation with the primary antibody (α-core C7-50 at a final concentration of 1 µg/mL or α-β-actin ACTBD11B7 at a final concentration 0,1 µg/mL; both purchased from Santa Cruz Biotechnologies, Santa Cruz, CA) was performed in blocking solution for 1 h at room temperature. Blots were washed five times for 10 min in washing solution (0.05% Tween 20 in PBS), incubated for 1 h with α-mouse horseradish peroxidase-conjugated secondary antibody in blocking solution (1∶5000, Sigma-Aldrich, St. Louis, MO), and washed as described above. Antibody-protein complexes were detected using the SuperSignal® West Femto Maximum Sensitivity Substrate Kit (Thermo Fischer Scientific Inc., MA).

### Luciferase assays

The Luciferase Assay System (Promega Madison, WI) was used for the quantification of luciferase reporter activity according to the manufacturer's protocol. In brief, electroporated Huh7.5 cells were resuspended in 10 ml complete DMEM, and 2 ml of the suspension was seeded per well in a six-well plate for harvesting at 4, 24, 48, 72, and 96 h after transfection (always in duplicates). To assay the luciferase activity, cells were washed once with PBS, and 150 µl of 1× cell culture luciferase lysis reagent was added. Cells were frozen immediately, and after having thawed, lysates were resuspended by pipetting. For each well, 3×50 µl lysate was mixed with 50 µl assay buffer (containing the luciferase substrate) measured for 5 s in a luminometer (Orion II Microplate Luminometer, Berthold Detection Systems, Pforzheim, Germany).

### Quantification of HCV core protein

HCV core protein in cell culture supernatants was quantified using the Ortho® HCV antigen ELISA test kit (Ortho Clinical Diagnostics, Tokyo, Japan). Cell culture supernatants were harvested at different time points, filtered through 0.45-µm-pore-size filters and kept at −80°C until the day of measurement. Depending on the construct and time of harvest, samples were diluted 1∶10 (or higher) and processed for ELISA according to the manufacturer's protocol. Colorimetric measurements were performed using a Sunrise colorimeter (Tecan Trading AG, Switzerland).

### RNA quantification by qRT-PCR

Viral RNA was isolated from virus-infected or electroporated cells using the Nucleo Spin RNAII kit (Macherey-Nagel, Düren, Germany) following the manufacturer's protocol. RNA concentration was determined by measuring the optical density at 260 nm. Twenty-five ng of the total RNA sample was used for quantitative qRT-PCR analysis with an Abbott m2000rt sequence detector system (Abbott Laboratories, Abbott Park, IL). HCV-specific qRT-PCRs were conducted in triplicate with the OneStep RT-PCR kit (QIAGEN, Hilden, Germany) using the following 5′ NTR-specific probe S-292, 5′-6-carboxyfluorescein- CCTGATAGGGTGCTTGCGAGTGCC -tetrachloro-6-carboxyfluorescein-3′; and primers; S-271, 5′- GCGAAAGGCCTTGTGGTACT-3′; and A-337, 5′- CACGGTCTACGAGACCTCCC -3′ (Biomers, Ulm, Germany). Reactions were performed in three stages under the following conditions: stage 1, 60 min at 55°C (reverse transcription); stage 2, 15 min at 95°C (heat inactivation of reverse transcriptase and activation of Taq polymerase); and stage 3, 40 cycles of 15 s at 95°C and 1 min 60°C (amplification). The total volume of the reaction mix was 15 µl, and it contained the following components: 2.66 µM 6-carboxy-X-rhodamine (passive reference), 4 mM MgCl_2_, 0.66 mM deoxynucleoside triphosphates, 0.266 µM HCV probe, 1 µM of each HCV primer, and 0.6 µl enzyme mix. The amount of HCV RNA was calculated by comparing it to serially diluted *in vitro* transcripts.

### Determination of virus titers in cell culture supernatants

Huh7.5 cells were seeded at a concentration of 1.5×10^4^ cells per well in a 96-well plate in a total volume of 200 µl complete DMEM. Twenty-four hours later, serial dilutions of virus containing supernatant were added (6 wells per dilution.) Three days later, cells were washed with PBS and fixed for 20 min with ice-cold methanol at −20°C. After three washes with PBS, NS5A was detected with a 1∶2000 dilution of the α-NS5A antibody 9E10 (kindly provided by C. Rice, The Rockefeller University, NY, USA) in PBS for 1 h at room temperature, supplemented with 5% BSA. Cells were washed again three times with PBS and bound primary antibodies were detected by incubation in PBS-5% BSA with goat α-mouse IgG-peroxidise conjugated antibody (Sigma-Aldrich, St. Louis, MO) at 1∶400 dilution. After 1 h incubation at room temperature, cells were washed three times with PBS; the Vector NovaRED substrate kit (Linaris Biologische Produkte GmbH, Wertheim, Germany) was used for detection of peroxidase. Virus titres (50% tissue culture infective dose per ml (TCID_50_/ml)) were calculated based on the method described by Spearman and Kärber.

### HCVpp production and infection of Huh7.5 cells

HCV pseudoparticles were generated by cotransfection of 293T cells with the jetPEI™ transfection reagent (Polyplus Transfection™, Illkirch, France) with equal amounts of CMV-driven expression cassette plasmids of the viral gps of the BHCV1 (pcDNA3.1ΔcE1E2-BHCV1 or mutants) or of the CG1b isolate (genotype 1b, plasmid kindly provided by Dr. T. Jake Liang) or an empty vector (no ENV) and the envelope-defective pNL4.3.Luc.R^−^E^−^ proviral HIV genome, according to the manufacturer's protocols. The supernatants were collected 48 h post transfection, cleared by passing them through 0.45-µm-pore-size filters. HIV p24 antigen content was assessed by using the commercially available p24 EIA – Innotest™ HIV Antigen mAb (Innogenetics, Gent, Belgium). Normalized supernatants (equal amounts of p24) were used to inoculate Huh7.5 cells for 6 h, seeded 18 h prior to inoculation in 12 well plates, 8×10^4^ cells/well. Cells were lysed 72 h post inoculation and luciferase activity was measured as described above.

### Site-directed mutagenesis

Site-directed mutagenesis to E1 or E2 glycoproteins in the pcDNA3.1ΔcE1E2-BHCV1 vector were introduced using the GeneTailor™ Site-Directed Mutagenesis System (Invitrogen, Carlsbad, CA) and primers designed according to the manufacturer's protocol.

## Supporting Information

Materials and Methods S1 **Protocols for HCV RNA extraction from serum and cloning procedures are provided at this section.**
(DOC)

Table S1 **Primers combinations for the cloning of the BHCV1 isolate.**
(PDF)

Table S2 **Primers combinations for the cloning of the JFH1 part.**
(PDF)

Table S3 **List of forward (sense) primers.**
(PDF)

Table S4 **List of reverse (antisense) primers.**
(PDF)

## References

[1] Simmonds P, Bukh J, Combet C, Deleage G, Enomoto N (2005). Consensus proposals for a unified system of nomenclature of hepatitis C virus genotypes.. Hepatology.

[2] Williams R (2006). Global challenges in liver disease.. Hepatology.

[3] Choo QL, Kuo G, Weiner AJ, Overby LR, Bradley DW (1989). Isolation of a cDNA clone derived from a blood-borne non-A, non-B viral hepatitis genome.. Science.

[4] Bartenschlager R, Ahlborn-Laake L, Mous J, Jacobsen H (1994). Kinetic and structural analyses of hepatitis C virus polyprotein processing.. J Virol.

[5] Grakoui A, McCourt DW, Wychowski C, Feinstone SM, Rice CM (1993). Characterization of the hepatitis C virus-encoded serine proteinase: determination of proteinase-dependent polyprotein cleavage sites.. J Virol.

[6] Kolykhalov AA, Agapov EV, Blight KJ, Mihalik K, Feinstone SM (1997). Transmission of hepatitis C by intrahepatic inoculation with transcribed RNA.. Science.

[7] Yanagi M, Purcell RH, Emerson SU, Bukh J (1997). Transcripts from a single full-length cDNA clone of hepatitis C virus are infectious when directly transfected into the liver of a chimpanzee.. Proc Natl Acad Sci U S A.

[8] Lohmann V, Korner F, Koch J, Herian U, Theilmann L (1999). Replication of subgenomic hepatitis C virus RNAs in a hepatoma cell line.. Science.

[9] Bartosch B, Dubuisson J, Cosset FL (2003). Infectious hepatitis C virus pseudo-particles containing functional E1-E2 envelope protein complexes.. J Exp Med.

[10] Hsu M, Zhang J, Flint M, Logvinoff C, Cheng-Mayer C (2003). Hepatitis C virus glycoproteins mediate pH-dependent cell entry of pseudotyped retroviral particles.. Proc Natl Acad Sci U S A.

[11] Lindenbach BD, Evans MJ, Syder AJ, Wolk B, Tellinghuisen TL (2005). Complete replication of hepatitis C virus in cell culture.. Science.

[12] Pietschmann T, Kaul A, Koutsoudakis G, Shavinskaya A, Kallis S (2006). Construction and characterization of infectious intragenotypic and intergenotypic hepatitis C virus chimeras.. Proc Natl Acad Sci U S A.

[13] Wakita T, Pietschmann T, Kato T, Date T, Miyamoto M (2005). Production of infectious hepatitis C virus in tissue culture from a cloned viral genome.. Nat Med.

[14] Zhong J, Gastaminza P, Cheng G, Kapadia S, Kato T (2005). Robust hepatitis C virus infection in vitro.. Proc Natl Acad Sci U S A.

[15] Moradpour D, Penin F, Rice CM (2007). Replication of hepatitis C virus.. Nat Rev Microbiol.

[16] Pietschmann T, Zayas M, Meuleman P, Long G, Appel N (2009). Production of infectious genotype 1b virus particles in cell culture and impairment by replication enhancing mutations.. PLoS Pathog.

[17] Kato T, Date T, Miyamoto M, Furusaka A, Tokushige K (2003). Efficient replication of the genotype 2a hepatitis C virus subgenomic replicon.. Gastroenterology.

[18] Delgrange D, Pillez A, Castelain S, Cocquerel L, Rouille Y (2007). Robust production of infectious viral particles in Huh-7 cells by introducing mutations in hepatitis C virus structural proteins.. J Gen Virol.

[19] Gottwein JM, Scheel TK, Hoegh AM, Lademann JB, Eugen-Olsen J (2007). Robust hepatitis C genotype 3a cell culture releasing adapted intergenotypic 3a/2a (S52/JFH1) viruses.. Gastroenterology.

[20] Gottwein JM, Scheel TK, Jensen TB, Lademann JB, Prentoe JC (2009). Development and characterization of hepatitis C virus genotype 1–7 cell culture systems: role of CD81 and scavenger receptor class B type I and effect of antiviral drugs.. Hepatology.

[21] Kaul A, Woerz I, Meuleman P, Leroux-Roels G, Bartenschlager R (2007). Cell culture adaptation of hepatitis C virus and in vivo viability of an adapted variant.. J Virol.

[22] Yi M, Ma Y, Yates J, Lemon SM (2007). Compensatory mutations in E1, p7, NS2, and NS3 enhance yields of cell culture-infectious intergenotypic chimeric hepatitis C virus.. J Virol.

[23] Yi M, Villanueva RA, Thomas DL, Wakita T, Lemon SM (2006). Production of infectious genotype 1a hepatitis C virus (Hutchinson strain) in cultured human hepatoma cells.. Proc Natl Acad Sci U S A.

[24] Silberstein E, Mihalik K, Ulitzky L, Plant EP, Puig M (2010). Persistent growth of a human plasma-derived hepatitis C virus genotype 1b isolate in cell culture.. PLoS Pathog.

[25] Blight KJ, McKeating JA, Rice CM (2002). Highly permissive cell lines for subgenomic and genomic hepatitis C virus RNA replication.. J Virol.

[26] Koutsoudakis G, Herrmann E, Kallis S, Bartenschlager R, Pietschmann T (2007). The level of CD81 cell surface expression is a key determinant for productive entry of hepatitis C virus into host cells.. J Virol.

[27] Friebe P, Boudet J, Simorre JP, Bartenschlager R (2005). Kissing-loop interaction in the 3′ end of the hepatitis C virus genome essential for RNA replication.. J Virol.

[28] Thomson M, Nascimbeni M, Gonzales S, Murthy KK, Rehermann B (2001). Emergence of a distinct pattern of viral mutations in chimpanzees infected with a homogeneous inoculum of hepatitis C virus.. Gastroenterology.

[29] Tamura K, Nei M (1993). Estimation of the number of nucleotide substitutions in the control region of mitochondrial DNA in humans and chimpanzees.. Mol Biol Evol.

[30] Miyanari Y, Atsuzawa K, Usuda N, Watashi K, Hishiki T (2007). The lipid droplet is an important organelle for hepatitis C virus production.. Nat Cell Biol.

[31] Kato N, Ootsuyama Y, Ohkoshi S, Nakazawa T, Sekiya H (1992). Characterization of hypervariable regions in the putative envelope protein of hepatitis C virus.. Biochem Biophys Res Commun.

[32] Yagnik AT, Lahm A, Meola A, Roccasecca RM, Ercole BB (2000). A model for the hepatitis C virus envelope glycoprotein E2.. Proteins.

[33] Jirasko V, Montserret R, Lee JY, Gouttenoire J, Moradpour D (2010). Structural and functional studies of nonstructural protein 2 of the hepatitis C virus reveal its key role as organizer of virion assembly.. PLoS Pathog.

[34] Appel N, Zayas M, Miller S, Krijnse-Locker J, Schaller T (2008). Essential role of domain III of nonstructural protein 5A for hepatitis C virus infectious particle assembly.. PLoS Pathog.

[35] Garcia-Retortillo M, Forns X, Feliu A, Moitinho E, Costa J (2002). Hepatitis C virus kinetics during and immediately after liver transplantation.. Hepatology.

[36] Rosen HR (2008). Transplantation immunology: what the clinician needs to know for immunotherapy.. Gastroenterology.

[37] Revie D, Braich RS, Bayles D, Chelyapov N, Khan R (2005). Transmission of human hepatitis C virus from patients in secondary cells for long term culture.. Virol J.

[38] Kato N, Shimotohno K (2000). Systems to culture hepatitis C virus.. Curr Top Microbiol Immunol.

[39] Vieyres G, Thomas X, Descamps V, Duverlie G, Patel AH (2010). Characterization of the envelope glycoproteins associated with infectious hepatitis C virus.. J Virol.

[40] Lopez-Labrador FX, Ampurdanes S, Forns X, Castells A, Saiz JC (1997). Hepatitis C virus (HCV) genotypes in Spanish patients with HCV infection: relationship between HCV genotype 1b, cirrhosis and hepatocellular carcinoma.. J Hepatol.

[41] van den Hoff MJ, Christoffels VM, Labruyere WT, Moorman AF, Lamers WH (1995). Electrotransfection with “intracellular” buffer.. Methods Mol Biol.

