# Supplementary material for: Cell Culture Replication of a Genotype 1b Hepatitis C Virus Isolate Cloned from a Patient Who Underwent Liver Transplantation
Source: PLoS One. 2011 Aug 24;6(8):e23587. doi: 10.1371/journal.pone.0023587 (PMC3160967; doi:10.1371/journal.pone.0023587)
Supplement: Materials and Methods S1 — Protocols for HCV RNA extraction from serum and cloning procedures are provided at this section. (DOC) [file pone.0023587.s001.doc]

# Cell culture Replication of a Genotype 1b Hepatitis C Virus Isolate Cloned From a Patient Who Underwent Liver Transplantation

George Koutsoudakis^1^, Sofia Perez-del-Pulgar^1^, Mairene Coto-Llerena^1^, Patricia Gonzalez^1^, Jakub Dragun^1^, Laura Mensa^1^, Gonzalo Crespo^1^, Miguel Navasa^1^, and Xavier Forns^1^

^1^: Liver Unit, Institut D'Investigacions Biomèdics August Pi i Sunyer, Centro de Investigación Biomédica en Red: Enfermedades Hepáticas y Digestivas, Hospital Clínic, Barcelona, Spain

# Supplementary Materials and Methods S1

## Isolation of HCV from patient serum and plasmids construction

Total RNA was extracted from 140 μl of serum by using the QIAamp viral RNA extraction kit (Qiagen, Düsseldorf, Germany) according to the manufacturer’s protocol. RNA was subjected to cDNA production with the High Capacity cDNA Reverse Transcription Kit (Applied Biosystems, Foster City, CA) and random hexameric primers according to the manufacturer’s conditions. cDNA reaction kept at -80 ^o^C and used as a template for the amplification of the HCV ORF (see below). In all PCR reactions described hereafter the platinum Taq Polymerase was used (Invitrogen, Carlsbad, CA).

## Cloning of the BHCV1 isolate

The 5´ NTR (nt 1-341) was cloned as a fragment of 377 nucleotides with a semi-nested PCR reaction with the 5´/3´ RACE kit (Roche, New York, NY) according to manufacturer´s protocol with a minor modification: random hexameric primers at a concentration of 12.5 μM were used at the initial step of the cDNA synthesis. A third PCR was followed with primers S-SwaI-Τ7 and the primer A-337 in order to add a *SwaI* restriction site for further cloning and the T7 promoter upstream of the 1 nucleotide of the 5´ NTR.

PCR primers for the amplification of HCV ORF were designed for conserved regions post alignment of several 1b isolates. Primers were re-named according to the nucleotide numbers of the BHCV1 isolate, including primer sequence. The HCV ORF was amplified in 5 fragments (nt 66-2662, nt 2226-4100, nt 2795-5434, nt 5327-6778, nt 6291-9421). Antisense primer A-9421 binds to a conserved region of the 3´ NTR, in order to cover the entire ORF. 2 μl of cDNA reaction were subjected to the first PCR reaction (total volume 20 μl) and PCR conditions consisted of 35 cycles each of denaturing 94 ^o^C for 30 seconds (sec), annealing for the first 5 cycles 50 ^o^C and the next 30 cycles 55 ^o^C for 15 sec, and extension at 68 ^o^C for 2 min. 5 μl of the first PCR reaction was used as DNA template for semi- or nested PCRs with the following modifications at the protocol: annealing for the 35 cycles at 55 ^o^C and a final step of 20 minutes at 68 ^o^C after the last amplification cycle in order to add 3'-A overhangs to each end of the PCR product. PCR reactions were electrophorised in agarose gels and products with the expected size were cut and purified with Qiagen Gel extraction kit (Qiagen, Düsseldorf, Germany). Purified PCRs were cloned into the pGEM-T easy vector (Promega, Madison, WI) and sequenced. The consensus sequence of 4 isolates was adopted for each region.

For the addition of the H77 3´ NTR to the genome, the fragment 8837-9421 was amplified directly from the cDNA and used as a template for a PCR reaction with the primers S-9022 & A-NS5B-3´NTR. A second PCR was done with template a plasmid containing the H77 3´ NTR and primers S-NS5B-3´NTR & A-HindIII-SbfI. Both PCRs were cleaned with agarose gel electrophoresis and subjected to a combined PCR with the primers S-9022 & A-HindIII-SbfI. Final combined PCR product was cleaned again by agarose gel electrophoresis and cloned also into the pGEM-T easy vector.

Full-length isolate was reconstructed by successive ligations to the initial pGEM-T easy vector containing the 5´ NTR fragment of the following fragments: An *AgeI-SbfI* fragment from the pGEM-T easy vector containing the 66-2662 fragment, a *MfeI-SbfI* fragment from the 2226-4100 vector, a *SfiI-SbfI* fragment from the 2795-5434 vector, a *FseI-SbfI* fragment from the 5327-6778 vector, a *PstI-SbfI* fragment from the 6291-9421vector and a *KpnI-SbfI* fragment from the vector contained the 3´ NTR. Finally, the whole full-length genome was re-sequenced and cloned into a puC vector, generating the plasmid puC-Barcelona HCV1 (pUC-BHCV1). The list of the primers combinations used for the cloning of the BHCV1 isolate is presented on the table S1.

## Construction of subgenomic replicons with luciferase reporter

For the cloning of a plasmid containing the subgenomic replicon with luciferase reporter, 3 fragments were cloned into the puC-BHCV1 vector opened with *AgeI-SfiI* restriction enzymes. Fragment 1: a PCR fragment with DNA template the puC-BHCV1 plasmid and primers S-66 & A-389-NotI, cut with *AgeI-NotI*. Fragment 2: a PCR fragment with DNA template a plasmid containing the *firefly luciferase* gene, sense primer containing a silent mutation inactivating the *XbaI* site S-Luc-NotI and antisense primer A-Luc-XhoI, cut with *NotI-XhoI*. Fragment 3: a first PCR was done for the amplification of the EMCV IRES with a plasmid containing this element and primers introducing the *XhoI* restriction site at the 5´ of the gene and at the 3´ fused to the NS3 protein of the BHCV1 isolate introducing a start codon; S-EI-XhoI & A-EI-NS3. A second PCR was done with template the puC-BHCV1 plasmid and primers S-EI-NS3 & A-4233. Purified PCRs were used as a template to a combination PCR in which DNA were fused to one product with primers S-EI-XhoI & A-4233. Combined PCR product was purified by agarose gel electrophoresis, cut with *XhoI-SfiI* and used further for ligation. Final vector puC-BHCV1–subgenomic replicon luciferase or puC-BHCV1-SGR-Luc was re-sequenced.

## Deletion of the GDD motif of the BHCV1 isolate

For the construction of a ΔGDD negative control variant of the puC-BHCV1 and puC-BHCV1-SGR-Luc plasmids two PCR reactions were done with DNA template the puC-BHCV1 plasmid and primers for the first PCR S-7039 & A-ΔGDD-BHCV1 and for the second S-ΔGDD-BHCV1 & A-HindIII-SbfI. The two PCRs were combined to one with primers S-7039 and A-HindIII-SbfI and the combined PCR product was agarose gel electrophorised, cut with *AscI-XbaI* restriction enzymes and inserted to the puC-BHCV1 and puC-BHCV1-SGR-Luc vectors previously linearized with same enzymes.

## Construction of ΔcE1E2 expression vector for the generation of HCVpp (pcDNA3.1-ΔcE1E2/BHCV1)

A cassette encompassing a start codon followed by the last 21 aa of core, E1 and E2 genes and a stop codon was amplified from the puc-BHCV1 vector by PCR and primers S-EcoRI-21C-BHCV1 & A-E2-Stop-XbaI-BHCV1, cut with the *EcoRI* and *XbaI* restriction enzymes and cloned to the pcDNA3.1 vector previously linearized at the *EcoRI* –*XbaI* sites.

## Amplification of the JFH1 part of the BHCV1/JFH1 virus

For the amplification of the JFH1 part of the BHCV1/JFH1 chimeric virus the same protocol was followed like in the case of the BHCV1 virus, with 7 distinct semi-nested PCRs. The list of the primers combinations used for the cloning of the JFH1 isolate is presented on the table S2. The exact sequence of primers is presented on the final tables with all primers used in this study.
