# Supplementary material for: Cell Culture Replication of a Genotype 1b Hepatitis C Virus Isolate Cloned from a Patient Who Underwent Liver Transplantation
Source: PLoS One. 2011 Aug 24;6(8):e23587. doi: 10.1371/journal.pone.0023587 (PMC3160967; doi:10.1371/journal.pone.0023587)
Supplement: Table S1 — Primers combinations for the cloning of the BHCV1 isolate. (PDF) [file pone.0023587.s002.pdf]

Supplementary Table S1. Primers´ combinations for the cloning of the BHCV1 isolate

Fragment (nt)	Primers	
	1st PCR	2nd PCR (nested or semi-nested)
1-377	Oligo d(T)-anchor primer[1] & A-337	PCR anchor primer[1] & A-337
66-2662	S-57 & A-2762	S-66 & A-2662
2226-4100	S-1740 & A-4128	S-2226 & A-4100
2795-5434	S-2784 & A-5480	S-2795 & A-5434
5327-6778	S-5319 & A-6793	S-5327 & A-6778
6291-9421	S-6282 & A-9421	S-6291 & A-9421
8837-9421	S-8816 & A-9421	S-8837 & A-9421

[1]: Primers derived by the 5´/3´ RACE kit (Roche, Cat. No. 03353621001)
