# Supplementary material for: Cell Culture Replication of a Genotype 1b Hepatitis C Virus Isolate Cloned from a Patient Who Underwent Liver Transplantation
Source: PLoS One. 2011 Aug 24;6(8):e23587. doi: 10.1371/journal.pone.0023587 (PMC3160967; doi:10.1371/journal.pone.0023587)
Supplement: Table S2 — Primers combinations for the cloning of the JFH1 part. (PDF) [file pone.0023587.s003.pdf]

Supplementary Table S2. Primers´ combinations for the cloning of the JFH1 part

Fragment (nt)	Primers	
	1st PCR	2nd PCR (nested or semi-nested)
2226-3943	S-2130 & A-3952-2a	S-2226 & A-3952-2a
3911-5332	S-3451-2a & A-5341-2a	S-3920-2a & A-5341-2a
5232-6311	S-4601-2a & A-6320-2a	S-5241-2a & A-6320-2a
6084-7013	S-5641-2a & A-7022-2a	S-6093-2a & A-7022-2a
6512-8031	S-6093-2a & A-8040-2a	S-6521-2a & A-8040-2a
7944-8827	S-7490-2a & A-8844-2a	S-7952-2a & A-8844-2a
8755-9430	S-8417-2a & A-9439-2a	S-8764-2a & A-9439-2a
