# Supplementary material for: Cell Culture Replication of a Genotype 1b Hepatitis C Virus Isolate Cloned from a Patient Who Underwent Liver Transplantation
Source: PLoS One. 2011 Aug 24;6(8):e23587. doi: 10.1371/journal.pone.0023587 (PMC3160967; doi:10.1371/journal.pone.0023587)
Supplement: Table S3 — List of forward (sense) primers. (PDF) [file pone.0023587.s004.pdf]

Supplementarty Table S3. List of forward (sense) primers

Name	Sequence (5′→ 3′)
S-SwaI-T7	AGAATTTAAATTAATACGACTCACTATAGCCAGCCCCCGATTGGGGGCGA
S-NS5B-3′NTR	CAACCGATGAACGGGGAGCTAAACACTCCAGGCCAA
S-57	ACTGTCTTCACGCAGAAAGCGTCTAGCCAT
S-66	ACGCAGAAAGCGTCTAGCCATGGCGTTAG
S-1740	CAGGGATGGGGTCCCATCA
S-2226	TTCAAGGTTAGGATGTATGTGGG
S-2784	GAGATGGCTGCATCGTGCGGAGGCGC
S-2795	ATCGTGCGGAGGCGCGGTTTTCGTAG
S-5319	AGCACCTGGGTGCTGGTAGGCG
S-5327	GGTGCTGGTAGGCGGAGTCCTTG
S-6282	GATGTTTGGGACTGGATATGCACGGTGT
S-8816	ACTCAACTCCTGGCTAGGCAACAT
S-6291	GACTGGATATGCACGGTGTTGACTGACT
S-8837	TATCATCATGTATGCGCCCACCCT
S-66	ACGCAGAAAGCGTCTAGCCATGGCGTTAGT
S-Luc-NotI	TCAACGCGGCCGCATGGAAGACGCCAAAAACATAAAGGAAGGCCCGGCGCCATTCTATCCTCTTGAG
S-EI-XhoI	TTCTGACTCGAGCCTCTCCCTCCCCCCCCCCTAACGTTAC
S-EI-NS3	CGATGATACCATGGCGCCCATCACGGCCTATTCCCAAC
S-7039	TCCTGTGGCGGCACGAGATGGG
S-ΔGDD-BHCV1	CAGGACTGCACAATCTGTGAAAGTGCGGGAACCCAAGAGG
S-EcoRI-21C-BHCV1	GGTGGAATTCGCCACCATGGGTTGCTCTTTCTCTATCTTCCTCT
S-9022	CACTCCATAGTTACTCTCCAGGT
S-2130	TGTGGTTCGGGGCCTTGGTTGAC
S-3451-2a	CCAGCAAACACGAGGCCTCCTGG
S-3920-2a	GCCAAATCCATCGATTTCATCCCCGTTG
S-4601-2a	TACTATAGAGGGTTGGACGTCTC
S-5241-2a	CTATTACCAATGAGGTCACCCTCA
S-5641-2a	ACACATGTGGAACTTCATTAGC
S-6093-2a	AATGGATGAACAGGCTTATTGCCT
S-6521-2a	CAGGGGACCTTTCCTATCAATTGC
S-7490-2a	TCAGAGACAGGTTCCGCCTCCTCTA
S-7952-2a	CTGCAAGATCCAAGTATGGATTCG
S-8417-2a	ACTGCCATACACTCGCTGACTGAG
S-8764-2a	TTCCTCAAATGTGTCTGTGGCGTT
