# Supplementary material for: Cell Culture Replication of a Genotype 1b Hepatitis C Virus Isolate Cloned from a Patient Who Underwent Liver Transplantation
Source: PLoS One. 2011 Aug 24;6(8):e23587. doi: 10.1371/journal.pone.0023587 (PMC3160967; doi:10.1371/journal.pone.0023587)
Supplement: Table S4 — List of reverse (antisense) primers. (PDF) [file pone.0023587.s005.pdf]

Supplementarty Table S4. List of reverse (antisense) primers

Name	Sequence (5′→ 3′)
A-NS5B-3′NTR	AGCTCCCCGTTCATCGGTTGGGGAGCAGATAAATGC
A-HindIII-SbfI	GCCAAGCT TGCATGCCTGCAGGTCGA
A-389-NotI	AATCATGCGGCCGCGTTGATGTTACGCTTGGTTTTTCTT
A-Luc-XhoI	AGAGGCTCGAGTTACAATTTGGACTTTCCGCCCTTCTTGG
A-EI-NS3	TGATGGGCGCCATGGTATCATCGTGTTTTTCAAAGG
A-ΔGDD-BHCV1	ACTTTCACAGATTGTGCAGTCCTGGAGCTTCGCAGCTCG
A-E2-Stop-XbaI-BHCV1	CTAGTCTAGATCAAGCCTCAGCTTGAGCTATCAGCAG
A-337	CACGGTCTACGAGACCTCCC
A-2762	TGGTGGTAACGCCAGCAGGA
A-4128	AGGTGGCAACGGACGGGTTCAGGA
A-4100	CACCTTGTACCCTTGGGCTGCAT
A-4233	ATCAGCATGCCTCGTGACCAAGTA
A-5480	GCACTCTTCCATCTCATCGAACTC
A-5434	CTGGTAGAGGACTTCCCTGTCGG
A-6793	ACCTGGAATGTGACCTCCTCCCGTAGG
A-9421	AAAGGGAATGGCCTATTGGC
A-3952-2a	TGTCTCAACGGGGATGAAATCGAT
A-5341-2a	AGCTAGGACCCACGTGCTGGTCAT
A-6320-2a	CTGTCAAGATGGTGCAAACCCAGTC
A-7022-2a	CGTCATAGGTGTTGCTGTGGGTGGTGC
A-8040-2a	TCCTTCCACACGGACTTGATGTG
A-8844-2a	AGTGGTTGGGTCTCTGGTCAGGTA
A-9439-2a	CCGAGCGGGGAGTAGGAAGAGGCC
